# Gut microbiota landscape and potential biomarker identification in female patients with systemic lupus erythematosus using machine learning

**DOI:** 10.3389/fcimb.2023.1289124

**Published:** 2023-12-19

**Authors:** Wenzhu Song, Feng Wu, Yan Yan, Yaheng Li, Qian Wang, Xueli Hu, Yafeng Li

**Affiliations:** ^1^ School of Public Health, Shanxi Medical University, Taiyuan, Shanxi, China; ^2^ Department of Nephrology, Shanxi Provincial People's Hospital (Fifth Hospital) of Shanxi Medical University, Taiyuan, China; ^3^ Shanxi Provincial Key Laboratory of Kidney Disease, Taiyuan, Shanxiuan, China; ^4^ Department of Nephrology, Hejin People’s Hospital, Yuncheng, Shanxi, China; ^5^ Core Laboratory, Shanxi Provincial People's Hospital (Fifth Hospital) of Shanxi Medical University, Taiyuan, China; ^6^ Academy of Microbial Ecology, Shanxi Medical University, Taiyuan, China

**Keywords:** systemic lupus erythematosus, machine learning, elastic net, Boruta, gut microbiota

## Abstract

**Objectives:**

Systemic Lupus Erythematosus (SLE) is a complex autoimmune disease that disproportionately affects women. Early diagnosis and prevention are crucial for women’s health, and the gut microbiota has been found to be strongly associated with SLE. This study aimed to identify potential biomarkers for SLE by characterizing the gut microbiota landscape using feature selection and exploring the use of machine learning (ML) algorithms with significantly dysregulated microbiotas (SDMs) for early identification of SLE patients. Additionally, we used the SHapley Additive exPlanations (SHAP) interpretability framework to visualize the impact of SDMs on the risk of developing SLE in females.

**Methods:**

Stool samples were collected from 54 SLE patients and 55 Negative Controls (NC) for microbiota analysis using 16S rRNA sequencing. Feature selection was performed using Elastic Net and Boruta on species-level taxonomy. Subsequently, four ML algorithms, namely logistic regression (LR), Adaptive Boosting (AdaBoost), Random Forest (RF), and eXtreme gradient boosting (XGBoost), were used to achieve early identification of SLE with SDMs. Finally, the best-performing algorithm was combined with SHAP to explore how SDMs affect the risk of developing SLE in females.

**Results:**

Both alpha and beta diversity were found to be different in SLE group. Following feature selection, 68 and 21 microbiota were retained in Elastic Net and Boruta, respectively, with 16 microbiota overlapping between the two, i.e., SDMs for SLE. The four ML algorithms with SDMs could effectively identify SLE patients, with XGBoost performing the best, achieving Accuracy, Sensitivity, Specificity, Positive Predictive Value, Negative Predictive Value, and AUC values of 0.844, 0.750, 0.938, 0.923, 0.790, and 0.930, respectively. The SHAP interpretability framework showed a complex non-linear relationship between the relative abundance of SDMs and the risk of SLE, with *Escherichia_fergusonii* having the largest SHAP value.

**Conclusions:**

This study revealed dysbiosis in the gut microbiota of female SLE patients. ML classifiers combined with SDMs can facilitate early identification of female patients with SLE, particularly XGBoost. The SHAP interpretability framework provides insight into the impact of SDMs on the risk of SLE and may inform future scientific treatment for SLE.

## Introduction

Advancements in medical technology and increasing awareness of health issues have brought a growing focus on women’s health, given their unique physiological structure and functions (Qiao et al., 2021). Systemic lupus erythematosus (SLE) is a chronic autoimmune disease with high incidence, frequent relapses, and a generally poor prognosis, particularly affecting women between the ages of 20 and 40 (Tsang and Bultink, 2021; Lazar and Kahlenberg, 2023). It exhibits a wide range of clinical manifestations, ranging from mild cutaneous issues to severe organ failure and complications during pregnancy (Barber et al., 2021). Besides, individuals with SLE face an increased risk of conditions like atherosclerosis, thrombosis, arterial inflammation, and vascular spasms compared to the general population (Samuelsson et al., 2021).Tragically, SLE ranks among the leading causes of death in young women (Yen and Singh, 2018), with a significantly elevated mortality rate.

In the USA, a meta-analysis of over 26,000 female SLE patients revealed a mortality rate 2.6 times higher than the general population (Lee et al., 2016). In Asia, the annual incidence ranges from 2.8 to 8.6 cases per 100,000 person-years, with a prevalence varying from 26.5 to 103 cases per 100,000 individuals (Chiu and Lai, 2010; Zou et al., 2014; Barber et al., 2021). The current treatment for SLE primarily involves glucocorticoids and immunosuppressants. While standard therapy is effective to some extent, it comes with severe side effects and is not suitable for long-term use (Golder and Tsang, 2020). Also, until now, the diagnosis of SLE is primarily based on clinical assessment, although there are a few instances where serologic tests show negative results. There are no specific diagnostic criteria for SLE, and diagnosis is frequently made using classification criteria, albeit with notable limitations (Fanouriakis et al., 2021). Since women with SLE often experience more severe symptoms and organ damage than men, early diagnosis and prevention are essential for women’s health, and identifying new biomarkers associated with SLE is of great clinical significance.

Studies have shown a close relationship between intestinal flora and the occurrence of SLE, with significant differences in gut microbiota composition and metabolites in SLE patients compared to healthy individuals (Hevia et al., 2014). A study found that the *Firmicutes*/*Bacteroidetes* ratio in SLE patients was significantly lower than that in healthy subjects, which was confirmed by quantitative PCR analysis. Besides, another study showed that *Proteobacteria* increased and *Ruminococcaceae* decreased in SLE patients in different regions of Heilongjiang (Wei et al., 2019). Additionally, there are significant differences in the levels of metabolites in the gut microbiota of SLE patients compared to healthy individuals, especially aromatic amino acids and phosphatidylinositol (Robinson et al., 2021).

Given the close relationship, constructing a model with significantly dysregulated microbiotas (SDMs) for SLE would make its early identification possible. Yet, traditional regression models, which are determined by maximum likelihood estimation, have not made much progress in disease auxiliary diagnosis (Song et al., 2022). Notably, machine learning (ML), a research hotspot in the field of life sciences, gains ground in various diseases, including cardiovascular and cerebrovascular diseases (Zheng et al., 2021), kidney diseases (Belur Nagaraj et al., 2020), tumours (Sammut et al., 2022), neurological diseases (Boutet et al., 2021), immune diseases (Chen et al., 2021). Therefore, the utilization of ML techniques for the diagnosis of SLE based on gut microbiota is of great interest. However, there is currently a relative lack of research on this topic. Thus, ML could be adopted to identify crucial microbiota that may be intimately associated with the onset and progression of SLE. Building multiple algorithms utilizing these significant microbiotas as predictive factors to recognize SLE may potentially offer novel insights and value for the microbiota perspective of SLE diagnosis. Furthermore, interpretability serves as a critical supplement to ML decisions, and by integrating interpretable techniques with the most effective ML algorithms and presenting the diagnostic rationale of the model to physicians through visualization methods, it could greatly facilitate the treatment of SLE, which helps promote women’s health.

Considering the mounting evidence linking gut microbiota dysbiosis to SLE, there is a growing interest in exploring the potential of using gut microbiota as a predictive indicator for early identification and prevention of SLE in female patients. To this end, the present study aimed to characterize the gut microbiota landscape using feature selection approaches, develop and validate ML algorithms for early identification of SLE in female patients. Additionally, we aimed to use interpretability techniques to visualize the predictive factor mechanism, shedding light on the complex relationships between gut microbiota and SLE risk occurrence, and providing a reference for the future scientific treatment of SLE.

## Materials and methods

### Participants

This is a cross-sectional study conducted between December 2018 and August 2019, in which 54 patients diagnosed with systemic lupus erythematosus (SLE) at the Second Hospital of Shanxi Medical University were enrolled. Additionally, 55 negative controls (NC) who had no history of rheumatic immune diseases or family history were also recruited at the Physical Examination Centre of Shanxi Provincial People’s Hospital. The inclusion criteria for the study were as follows: patients with primary SLE diagnosed by a rheumatologist and who met the 1997 classification criteria for SLE as revised by the American College of Rheumatology (Hochberg, 1997). Exclusion criteria included patients with other autoimmune diseases, those who had received immunosuppressant or antibiotic therapy in the past 2 months, those with incomplete clinical data, those who were pregnant, those with special dietary habits, and those with severe hepatic and renal insufficiency.

**Figure 1:**
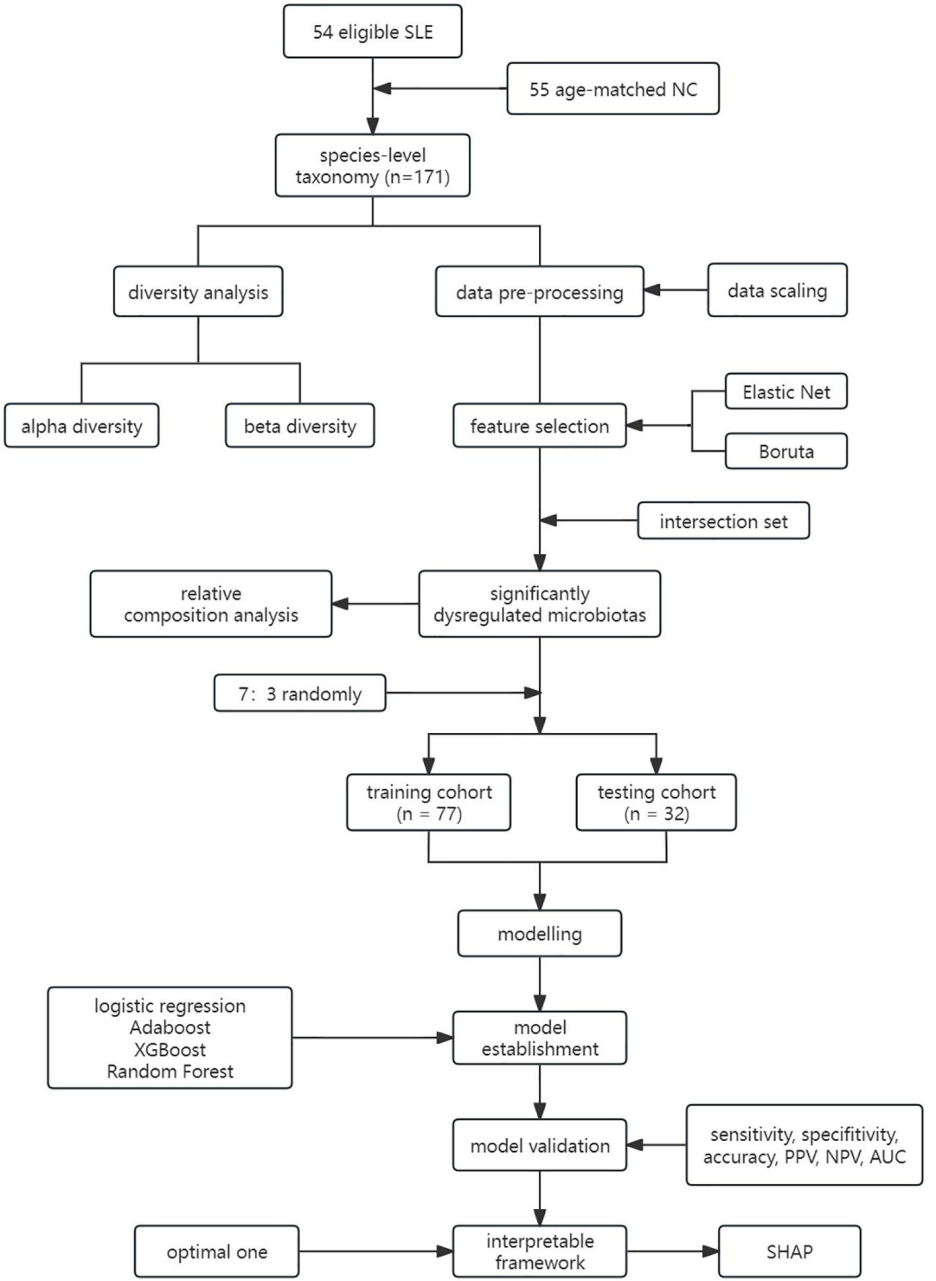
Workflow of this study. LR, Logistic Regression; LASSO, Least Absolute Shrinkage and Selection Operator; CART, Classification and Regression Tree; Adaboost, Adaptive Boosting; XGBoost, Extreme Gradient Boosting; RF, Random Forest; PPV, Positive Predicted Value; NPV, Negative Predicted Value; AUC, Area Under the Curve.

Ethics approval for the study was obtained from the Ethics Committee of the Second Hospital of Shanxi Medical University (Ethics No.: 2019-YX-107), and all patients provided informed consent. The study workflow is illustrated in **Figure 1**.

### Sample collection and experiments

Demographic information, including age and gender, was collected from study participants before the experiment. Stool samples were collected from both the SLE and NC groups after defecation during admission or health examination, and stored at -80°C in a protective solution using sterile cotton swabs for subsequent sequencing. The detection of microorganisms in the intestinal flora is based on the Illumina MiSeq sequencing platform, which employs bipartite sequencing technology to construct sequence libraries of small fragments that can be analyzed by sequencing. By splicing and filtering the Reads, clustering or noise reduction to obtain the Operational Taxonomic Unit (OTU), and then annotating and analyzing the abundance of the species, the species composition of the intestinal flora of the research object can be revealed.

The experiment procedures consist of DNA extraction and Illumina sequencing, Operational taxonomic unit (OTU) cluster analysis and species annotation and Bioinformatics analysis, as previously described (Xin et al., 2022).

According to the manufacturer’s instructions, we first removed the fecal specimens adsorbed with enteric flora from the cannula and extracted the total DNA of the microorganisms in the samples using the MagMAX™ Nucleic Acid Separation Kit; we tested the DNA quality of the samples by agar gel electrophoresis, NanoDrop One and Qubit 4 (Thermo Fisher Scientific); we extracted the DNA, and analyzed it by Phanta EVO Ultra The DNA was extracted and amplified by Phanta EVO Ultra Fidelity DNA Polymerase (Vazyme) to amplify the highly variable region of V3-V4 of the microbial 16S rRNA gene. The product was purified and recovered by FC magnetic bead kit (enlightenment). After purification, the concentration of the purified product was detected by Qubit 4, and its length distribution was detected on a Qsep100 Bioanalyzer, and the fragment length was basically in the range of 350-400 bp. Each PCR product was mixed homogeneously at the same final concentration and sequenced 75 bp bipartite using the Illumina MiSeq platform using MiSeq V2 reagent.

Pairs of raw data were acquired by sequencing using the Illumina Miseq platform, followed by preprocessing of the raw data, i.e., quality control, including low-quality filtering, and length filtering, to obtain high-quality sequences. When the single-end sequencing Read was less than 50 bp, the pair of Paired Reads was deleted.When the single-end sequence read contained bases (Q value< 5) less than 50% of the read length, the read was deleted.The above steps were performed in Fastp software.

In addition, host genes were deleted using Bowtie2 software, i.e., comparing them with the host sequence and filtering out reads that may be of host origin.Finally, quality-controlled Clean Reads were compared with microbial databases using Kraken2 software to obtain microbial data. In order to study the diversity information of microbial composition of the samples, the valid sequences of all samples were clustered into OTUs using Vsearch 2.4.4 with 97% similarity (Hu et al., 2022). The representative sequences were then analyzed by Silva128 database (http://www.arb-silva.de/) for species annotation and taxonomy.

Also, we discuss the α diversity and β diversity between the two groups. As for the β diversity, both Principal Co-ordinates Analysis (PCoA) based on the Bray-Curtis distance matrix and Permutation-based Multivariate Analysis of Variance (PERMANOVA) and Non-metric multidimensional scaling (NMDS) were employed to determine the significance of the difference (Franklin et al., 2022).

### Feature selection

Feature selection is a crucial step in data mining, which eliminates irrelevant and redundant features while preserving the original data’s value. It improves the data quality and reduces computational costs, enhancing the model’s generalization ability (Abbasi Mesrabadi et al., 2023). In the analysis of intestinal microbiota in SLE patients, characterizing microbiota data at the taxonomic level and removing redundant and irrelevant features is necessary to reduce noise. ML algorithms offer three feature selection methods: filtering, packaging, and embedding (Saha et al., 2022). This study uses embedded-based Elastic Net and packaging-based Boruta algorithms due to the differences in the subset of features selected by different methods.

The Elastic Net algorithm is a linear regression model that utilizes L1 and L2 regularization matrices. It inherits the sparsity of the LASSO method and the stability of the ridge regression L2 regularization. Elastic Net combines the two to obtain an optimal sparse model when cross-validating feature selection while compensating for the correlation between observed variables. Most feature selection methods seek the feature set that minimizes the model’s loss function. In contrast, Boruta selects a set of features relevant to the outcome. It is a wrapping algorithm for all relevant feature selections and identifies all features related to classification in the candidate features, determining the optimal subset of features (Kursa and Rudnicki, 2010; Kursa and Rudnicki, 2010).

### Model construction

To investigate the potential of the intestinal significantly dysregulated microbiota as a biomarker for SLE, four ML algorithms were utilized to establish the relationship between 16 SDMs and SLE. These algorithms include logistic regression (LR), adaptive boosting (AdaBoost), eXtreme gradient boosting (XGBoost), and Random Forest (RF).

LR is a generalized linear model that assumes a Bernoulli distribution for the dependent variable y. Logistic regression introduces the Sigmoid function to better handle nonlinear classification problems, unlike linear regression, which assumes a Gaussian distribution for y (LaValley, 2008; Meurer and Tolles, 2017). AdaBoost is a fundamental ML algorithm that assigns different weights to the bit error rate of the weak classifier to create a strong classifier (Sevinç, 2022). XGBoost is an enhanced algorithm that combines individual learners to efficiently construct parallel operations on the augmented tree (Davagdorj et al., 2020). By adding regular terms to the original function, it reduces the possibility of overfitting and accelerates the convergence speed (Ogunleye and Wang, 2020). RF is an ensemble learning algorithm that constructs multiple classification trees based on autonomously sampled training data. It selects independent variables to achieve decorrelation between trees, which in turn reduces the model variance. The final classification result is obtained by aggregating the results of multiple trees (Liu et al., 2022).

In summary, these algorithms were chosen for their ability to handle nonlinear classification problems, reduce overfitting, and achieve strong generalization ability.

### Model evaluation

This study employs various performance evaluation metrics for ML algorithm models, including Accuracy, Sensitivity, Specificity, Positive Predictive Value (PPV), Negative Predictive Value (NPV), and the receiver operating characteristic curve (ROC curve) with Area under the Curve (AUC).

### SHAP interpretability framework

SHAP is an additivity interpretation framework developed by Lundberg et al. and is based on the ideas of game theory (Li et al., 2023). For any individual, the prediction model outputs a prediction, and the SHAP framework assumes that each feature is a “contributor” to the target prediction and assigns Shapley values to them. The sum of the cumulative Shapley value of the target prediction and the average prediction value gives the contribution of all features of the individual.

The shapley value for a particular feature $x_{j}$ represents the average marginal contribution of that feature to the prediction. The difference between the predictions with and without feature $x_{j}$ is obtained by calculating the difference between the predictions with and without feature $x_{j}$ for all possible combinations, specifically by weighted summation with all possible combinations of features:

EQUATION 1


(1) $$\begin{array}{c} {\text{Φ}}_{j}\left(val\right)=\sum_{S\subseteq \left\{x_{1},\ldots ,x_{p}\right\}\backslash \left\{x_{j}\right\}}\frac{\left|S\right|!\left(p-\left|S\right|-1\right)!}{p!}\left(val\left(S\cup \left\{x_{j}\right\}\right)-val\left(S\right)\right) \end{array}$$

EQUATION 2


(2) $$\begin{array}{c} val\left(S\right)=\int \hat{f}\left(x_{1},\ldots ,x_{p}\right)d{\mathbb{P}}_{x\notin S}-E_{x}\left(\hat{f}\left(X\right)\right) \end{array}$$

x represents the feature value of the individual to be interpreted, $S\subseteq \left\{x_{1},\ldots ,x_{p}\right\}\backslash \left\{x_{j}\right\}$ is the subset of all features excluding $\left\{x_{j}\right\}$ , p is the number of features in the subset, $val\left(S\cup^{}\left\{x_{j}\right\}\right)$ denotes the prediction of all features in the subset that contains $\left\{x_{j}\right\}$ , val(S) is the prediction of the subset S that does not contain $\;\left\{x_{j}\right\}$ of all feature, $E_{x}\left(\hat{f}\left(X\right)\right)$ is the predicted expectation of all features, where $\frac{\left|S\right|!\left(p-\left|S\right|-1\right)!}{p!}$ is the weight of the subset S . For the understanding of the weights of subset S: p features have p !possibilities under any ordering, and after determining the subset S , p features have |S|!(p−|S|−1)! kinds of possibilities, so $\frac{\left|S\right|!\left(p-\left|S\right|-1\right)!}{p!}$ is the possible share of feature combinations for subset S .

Positive SHAP values (>0) indicate a feature positively affects the predicted value, while negative SHAP values (<0) indicate an adverse impact (Jiang et al., 2021). SHAP provides both global and local explanations. Global explanations calculate the average SHAP value of each feature on the entire dataset, while local explanations determine the SHAP value of each feature for a single sample. The SHAP framework is useful in understanding the mechanism behind predictive models and making informed decisions.

### Statistical analysis

The statistical analysis in this study was performed using R software (version 4.2.0). Continuous variables with normal distributions were reported as Mean ± Standard Deviation, while skewed variables were presented as median (interquartile range). Two-sample t-tests or Wilcoxon’s rank sum tests were utilized to draw statistical inferences. To build the ML models, random sampling was employed to split the dataset into training sets (70%) and test sets (30%), respectively. The level of significance was set at P< 0.05.

## Results

### Baseline characteristics

This study enrolled a total of 54 female patients diagnosed with SLE and 55 healthy women in the NC group. The mean age of SLE patients was 39.0 (32.0, 50.0) years, while the mean age of the NC group was 34.0 (28.0 to 53.0) years. The age difference was not statistically significant (P = 0.111), as shown in **Table 1**.

**Table 1:** Comparision of age in two groups.

Variable	NC (N=55)	SLE (N=54)	p
age	39.00 (32.00 to 50.00)	34.00 (28.00 to 53.00)	0.111

### Diversity analysis

**Figure 2:**
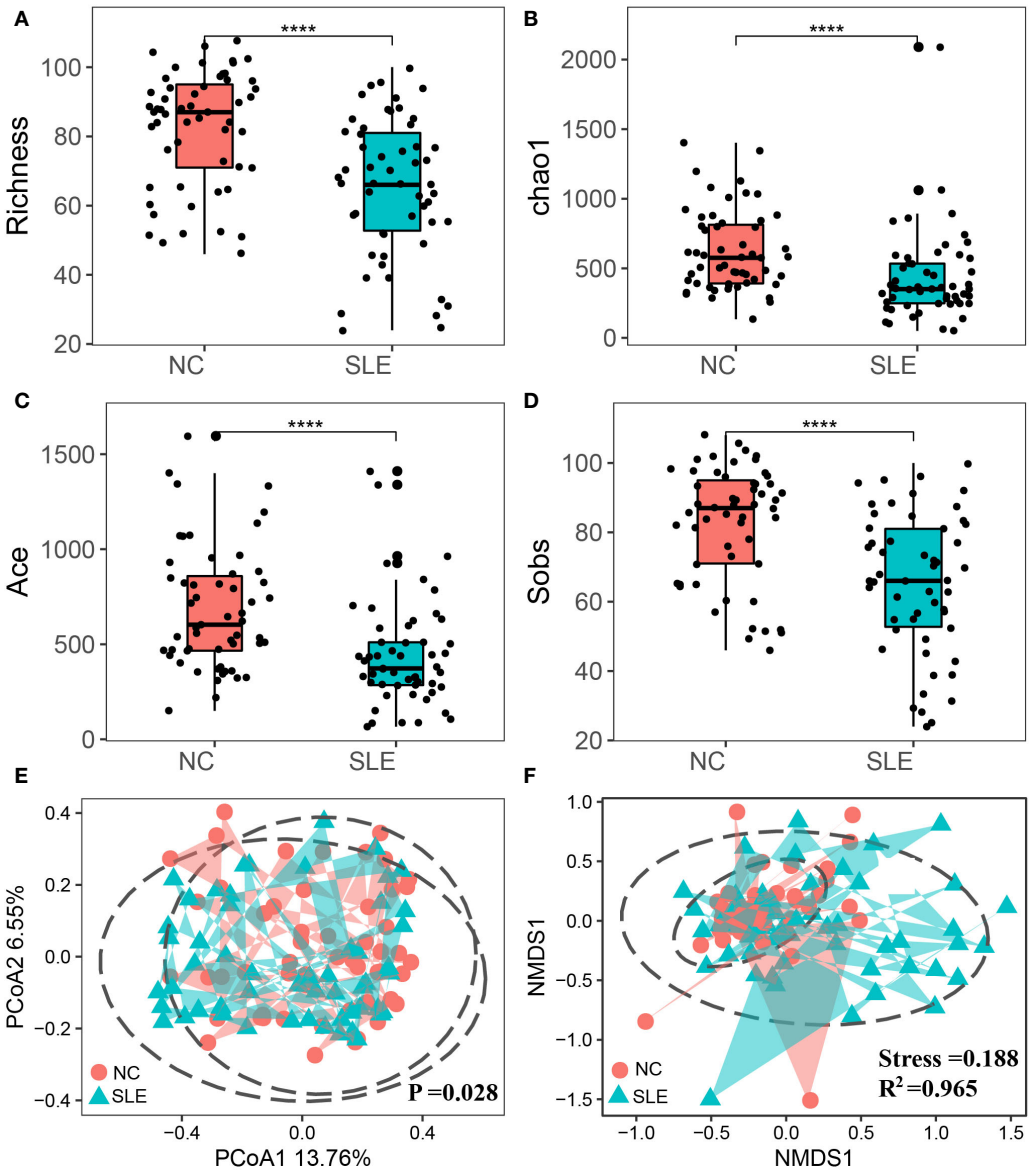
Comparison of alpha and beta diversity between SLE and NC groups. The points in the figure represent samples; *P<0.0001; **(A)** chao1 index; **(B)** Richness index; **(C)** Sobs index; **(D)** Shannon index; **(E)** Principal Co-ordinates Analysis; the closer the distance between samples, the more similar the species; **(F)** Non-metric multi-dimensional scale analysis, the closer the distance between samples, the higher the similarity between samples; Stress value<0.2 indicated that NMDS could explain the similarity of sample structure to some extent; The R^2^ was defined as the ratio of variance to the total variance of each group, and higher R^2^ values indicated a higher degree of explanation for sample differences between different groups.

This study employed alpha diversity to assess the richness and evenness of intestinal microbiota in SLE patients. The findings indicated that the Chao1 and Richness indices of the SLE group were significantly lower than those of the NC group (P< 0.001), indicating reduced bacterial Richness in the SLE group (**Figures 2A, B**). Furthermore, the Ace and Sobs indices of the SLE group were also significantly lower than those of the NC group (P< 0.05), suggesting an abnormal Evenness in the SLE group (**Figures 2C, D**).

Beta diversity was utilized to assess the structural composition similarity of intestinal flora. PCoA analysis revealed that Coordinate 1 accounted for 13.76% and Coordinate 2 occupied 6.55%. Also, the PERMANOVA analysis demonstrated that there were significant structural differences between the two groups (P = 0.028). Additionally, NMDS analysis indicated dissimilarity of species composition between the two groups, as demonstrated in **Figures 2E, F**.

### Feature selection

**Figure 3:**
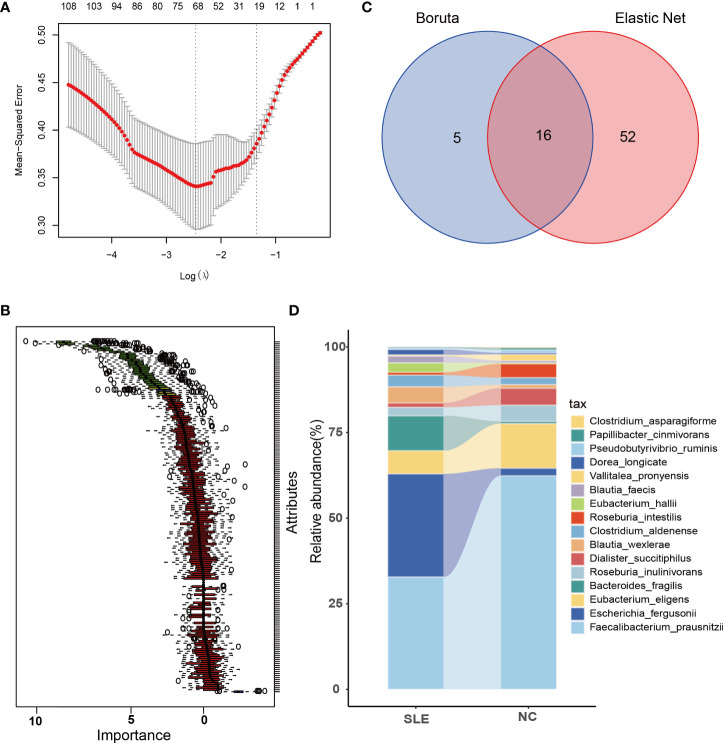
Feature selection and comparison of SDMs between the two groups. **(A)** When Log (Lamda) is taken the smallest, there are 68 bacteria left after using Elastic Net for feature selection; **(B)** There are 21 bacteria left after using Boruta for feature selection, the red part in the figure is the eliminated intestinal flora, and the green part is the remaining ones; **(C)** the intersected bacteria by using Elastic Net and Boruta, a total of 16, i.e. significantly dysregulated microbiotas (SDMs). **(D)** comparison of 16 SDMs in the two groups.

In total, there were 171 microbial species, which were considered for feature selection. To obtain a subset of highly correlated and non-redundant flora, Elastic Net and Boruta were utilized for feature selection. The parameters of Elastic Net for α equals 0.3 and for Lambda_min_ equals 0.085. Besides, the parameters employed for Boruta were as follows: doTrace=1, ntree=500, and maxRuns=350. The resulting flora subsets consisted of 68 and 21 features in Elastic Net and Boruta, respectively (**Figures 3A, B**). Among these subsets, 16 SDMs were identified (**Figure 3C**).

Among them, the abundance of *Faecalibacterium_prausnitzii*, *Eubacterium_eligens*, *Dialister_succitiphilus*, and *Roseburia_intestilis*, while *Roseburia_inulinivorans*, *Vallitalea_pronyensi*, *Pseudobutyrivibrio_ruminis*, *Papillibacter_cinmivorans*, and *Clostridium_asparagiforme* were comparatively higher in SLE group, while the abundance of *Escherichia_fergusonii* and *Bacteroides_fragilis* were higher in the SLE group, with *Blautia_wexlerae*, *Eubacterium_hallii*, *Blautia_faecis*, *Clostridium_aldenense*, and *Dorea_longicate* showing a slightly higher abundance in the SLE group as well (**Figure 3D**).

### Comparison of SDMs in training and test sets

**Figure 4:**
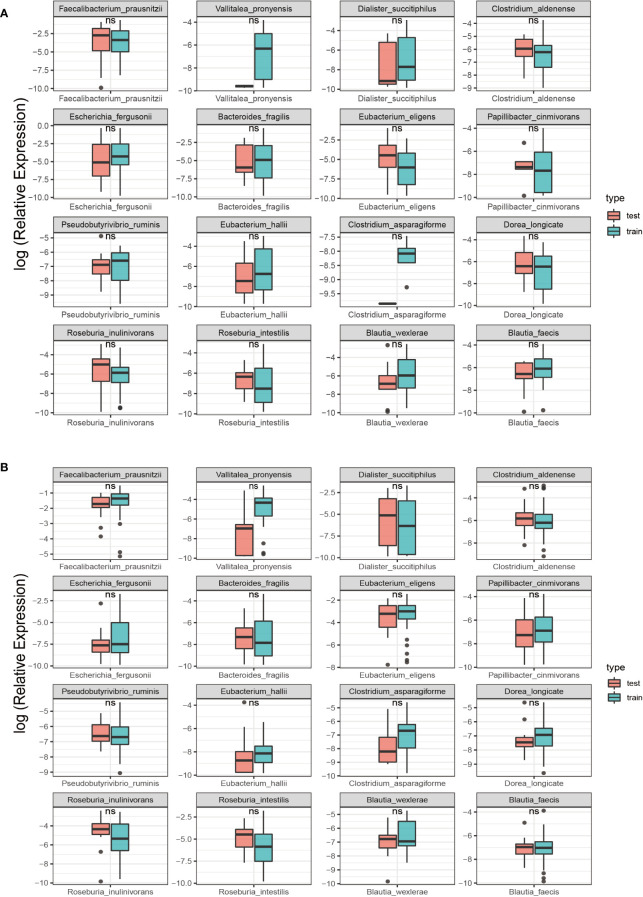
Comparison of SDMs between the training and test sets in the two groups. Red represents the testing set; Green represents the training set; ns, non-significant, P > 0.05; **: P< 0.01. **(A)** SLE group; **(B)** NC group.

This study randomly allocate the training and testing sets, achieving a balanced ratio of 39:38 for NC and SLE in the training set, and 16:16 in the testing set. This balanced ratio of 1:1 was implemented to mitigate any adverse impact on model training arising from class imbalance. Additionally, a rank sum test was conducted to ensure the comparability of microbiota in both sets and to facilitate an accurate evaluation of model performance. The findings revealed no statistically significant difference (P > 0.05) in the 16 SDMs between the groups in both sets, as demonstrated in **Figure 4**.

### Model evaluation

In this study, model performance evaluation was conducted using Accuracy, Sensitivity, Specificity, PPV, NPV and AUC. As shown in **Table 2**, all four ML algorithms showed commendable classification performance, with values exceeding 0.750 for all metrics. Specifically, LR achieved a value of 0.750 for each metric. AdaBoost achieved an Accuracy, Sensitivity, Specificity, PPV, and NPV of 0.844, 0.875, 0.813, 0.824, and 0.867, respectively. XGBoost exhibited metrics of 0.844, 0.750, 0.938, 0.923 and 0.930, while RF achieved metrics of 0.875, 0.750, 1.000, 1.000, and 0.800. AUC values were ranked as follows: XGBoost (0.930), RF (0.875), AdaBoost (0.844), and LR (0.750), demonstrating the high performance of the models.

**Table 2:** Model performance evaluation of four algorithms.

Algorithms	Accuracy	Sensitivity	Specificity	PPV	NPV	AUC
LR	0.750	0.750	0.750	0.750	0.750	0.750
Adaboost	0.844	0.875	0.813	0.824	0.867	0.844
XGBoost	0.844	0.750	0.938	0.923	0.790	0.930
RF	0.875	0.750	1.000	1.000	0.800	0.875

PPV, Positive Predicted Value; NPV, Negative Predicted Value.

### XGBoost-SHAP interpretable framework

**Figure 5:**
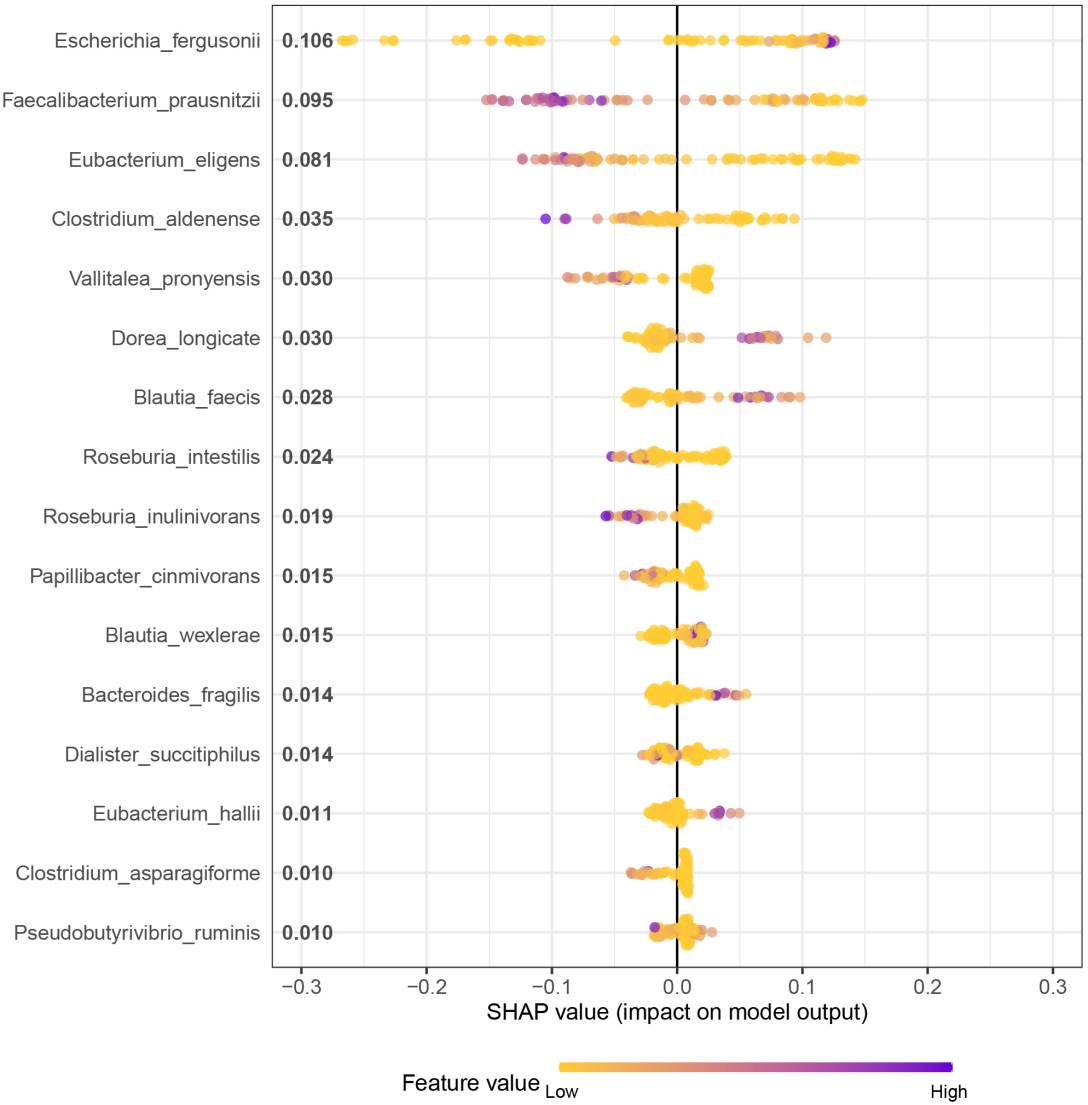
XGBoost-SHAP partial dependency plot Analysis for Distribution of Feature Importance in SLE occurrence risk.

This study employed the XGBoost algorithm in combination with the SHAP interpretability framework, owing to the former’s superior performance compared to other models. The SHAP values of 16 SDMs were outputted to obtain global and partial dependence plots for the SLE risk of disease. The SHAP global dependency plot in **Figure 5** ranks the importance of XGBoost algorithm variables and their respective positive and negative effects on SLE. The top 10 bacteria in terms of importance are *Escherichia_fergusonii*, *Faecalibacterium_prausnitzii*, *Eubacterium_eligens*, *Clostridium_aldenense*, *Vallitalea_pronyensis*, *Dorea_longicate*, *Blautia_faecis*, *Roseburia_intestilis*, *Roseburia_inulinivorans*, and *Papillibacter_cinmivorans*. The colour gradient from low to high represents the feature value from small to large, while the positive and negative SHAP values denote the correlation between the feature and the prediction result. For instance, a negative value for *Eubacterium_eligens* in a sample with a large value suggests a reduced risk of SLE. The SHAP value is mainly concentrated in the region with a positive SHAP value in the lower half of the colour gradient in the figure, indicating that the higher the relative expression of *Roseburia_intestilis*, the lower the risk of SLE.

**Figure 6:**
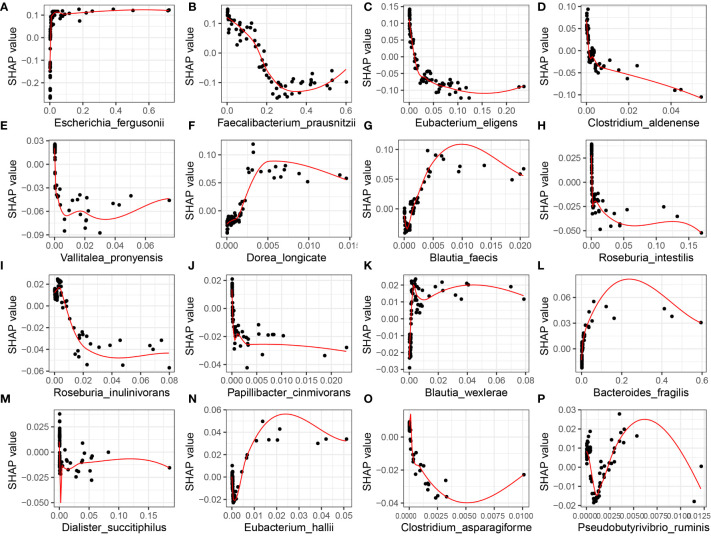
XGBoost-SHAP Global Dependence Plot Analysis for Distribution of Feature Importance in SLE occurrence risk. The distribution of the impact of each feature on the model is displayed in the figure, where each point corresponds to a sample and the colour of the point represents the magnitude of the variable value. The variable values are colour-coded from yellow to purple, corresponding to a high-to-low gradient. The x-axis represents relative abundance. **(A)** *Escherichia_fergusonii*; **(B)** *Faecalibacterium_prausnitzii*; **(C)** *Eubacterium_eligens*; **(D)** *Clostridium_aldenense*; **(E)** *Vallitalea_pronyensis*; **(F)** *Dorea_longicate*; **(G)** *Blautia_faecis*; **(H)** *Roseburia_intestilis*; **(I)** *Roseburia_inulinivorans*; **(J)** *Papillibacter_cinmivorans*; **(K)** *Blautia_wexlerae*; **(L)** *Bacteroides_fragilis*; **(M)** *Dialister_succitiphilus*; **(N)** *Eubacterium_hallii*; **(O)** *Clostridium_asparagiforme*; **(P)** *Pseudobutyrivibrio_ruminis*.

However, the SHAP global dependency plot is limited in its ability to accurately reveal the relationship between each feature and SLE. Therefore, we analyzed the SHAP partial dependency plot to better comprehend the impact of feature samples on SLE, as depicted in **Figure 6**. Each point in the plot represents the sample value of that feature, where the abscissa denotes the magnitude of the feature value and the ordinate represents the SHAP value of the corresponding feature. For *Faecalibacterium_prausnitzii*, the SHAP value indicates a trend of first decreasing and then rising, implying that the relative expression of the bacterium and the risk of SLE exhibit a complex nonlinear relationship, with a lower risk first and higher risk afterwards.

## Discussion

Women’s health is a crucial issue in contemporary society, and research indicates that gut microbiota plays a vital role in women’s health. However, the aetiology of SLE, a female-dominated immune disease, remains incompletely understood. This study aimed to explore the dysregulation of gut microbiota in SLE patients and identify significantly dysregulated microbiota in SLE patients in comparison with NC using ML algorithms.

In this study, we found significant differences in alpha and beta diversity between the SLE and NC groups, indicating gut microbiota dysregulation in SLE patients, which is consistent with prior findings in related immune diseases, such as autoimmune hepatitis (Wei et al., 2020), rheumatoid arthritis (Qiao et al., 2022), and primary dry syndrome (Siddiqui et al., 2016). The investigation identified 16 significantly dysregulated microbial taxa in SLE patients using Elastic Net and Boruta. We also revealed a complex relationship between the relative abundance of each bacterium and the risk of SLE using the SHAP interpretability framework.

Feature selection (Açıkoğlu and Tuncer, 2020) is a common data preprocessing method used in ML modelling. It reduces model complexity and improves accuracy by selecting a subset of features with strong discriminatory power. Among the significantly dysregulated microbial taxa, *Faecalibacterium_prausnitzii*, *Eubacterium_eligens*, and *Roseburia_intestilis* were found to be more abundant in the NC group, while *Escherichia_fergusonii* and *Bacteroides_fragilis* were more abundant in the SLE group. These microbial taxa are associated with gut health, systemic inflammation, and immune diseases. *Roseburia_intestilis* is a butyrate-producing bacterium that maintains gut health and alleviates systemic inflammation through the production of metabolites. It has the potential to improve atherosclerosis (Kasahara et al., 2018; Nie et al., 2021). *Faecalibacterium_prausnitzii* is one of the most common gut bacteria in healthy adults. Changes in its abundance may lead to the occurrence of immune diseases (Miquel et al., 2013; Lopez-Siles et al., 2017). *Eubacterium_eligens* plays an important role in intestinal inflammation. Studies have shown that its abundance is significantly reduced in patients with atherosclerosis (Liu et al., 2020) and hypertension (Nakai et al., 2021). *Escherichia_fergusonii* is a pathogenic bacterium that causes infections in humans and animals (Gaastra et al., 2014) and is overexpressed in the gut of non-alcoholic fatty liver disease patients (Xin et al., 2022). An increase in its abundance may contribute to the development of SLE. Previous studies have shown that the abundance of *Proteobacteria* increases, while *Ruminococcaceae* decreases in the gut microbiome of SLE patients (Hevia et al., 2014; Wei et al., 2019). This is not consistent with this study, which may be caused by regional differences.

The nonlinearity between gut microbiota and SLE has not been adequately modelled due to high dimensionality and significant inter-individual variability of gut microbiota (Qutrio Baloch et al., 2020). Recent advancements in artificial intelligence and big data have led to the emergence of data-driven ML algorithms in medical research (Ngiam and Khor, 2019). In this study, we divided the gut microbiota data into training and testing sets, with a 7:3 ratio, respectively. The feasibility of this approach was confirmed by the non-statistically significant differences of the 16 SDMs between the two datasets and a 1:1 SLE to NC ratio.

In this study, XGBoost showed the best performance, followed by RF. RF is a classifier that can be trained and used for prediction through multiple decision trees (Zhang et al., 2023). It is capable of adapting to complex datasets and improves the richness, generalizability, stability, and accuracy of the results by using random sampling (Yu and Zeng, 2022). This method can effectively avoid overfitting, and therefore, the overall performance of the RF was better than other algorithms, second only to XGBoost. Boosting is an effective ML technique that transforms basic weak classifiers into strong classifiers using ensemble algorithm theory to achieve better classification performance (Davagdorj et al., 2020). XGBoost demonstrated robustness and improved the model’s accuracy, avoiding overfitting and being less constrained by linear, collinear, and other issues compared to traditional models (Liang et al., 2021). Therefore, the performance of XGBoost was better than that of other algorithms.

Although sophisticated algorithms can potentially benefit patients by improving accuracy in clinical decision-making, their ambiguous decision-making processes create a “black box” dilemma. To address this issue, SHAP feature attribution analysis was employed to determine the influence of each attribute on the final model output, thereby improving the model’s interpretability. This study is the first to use SHAP to explore the impact of microbiota on SLE, revealing how changes in the relative abundance of SDMs affect the risk of SLE. For example, an increase in the relative abundance of *Escherichia_fergusonii* was found to increase the risk of SLE, while the relationship between *Faecalibacterium_prausnitzii* and SLE risk was complex and non-linear. However, the risk of SLE increased after a certain reduction in the relative abundance of *Faecalibacterium_prausnitzii*, suggesting that intestinal flora can be used as a dynamic detection index for SLE to identify high-risk groups. Clinical trials are necessary to validate the model and guide clinicians in conducting reasonable interventions on the flora that increase the risk of SLE, thus providing a new direction for individualized treatment of SLE.

While there is a large body of research exploring the relationship between the gut microbiota and SLE (Chen et al., 2022; Mohd et al., 2023; Toumi et al., 2023), it is important to note that our study focused specifically on female participants. This thoughtful choice was made due to the higher prevalence of SLE in women, making our study more gender-specific and relevant to the population most affected by the disease. This unique approach allowed us to gain insight into how SLE manifests itself in more common groups. In addition, our study first introduced ML-based SHAP to investigate how specific microbial communities influence the occurrence of SLE. This innovative approach provides a more detailed understanding of the key microbial factors contributing to the development of SLE, allowing us to analyze the complex relationship between gut microbiota and SLE more comprehensively.

The study has several limitations. Firstly, the sample size was small, and the data were collected from only one hospital in Shanxi province, which may limit the generalizability of the results. To enhance the robustness of our findings, future studies should aim to include larger sample sizes from different regions to more fully control for potential confounders and to improve the external validity of the results. Additionally, the study only controlled for age, and other confounders may have affected the results. Future research should include larger sample sizes from multiple regions to better control for other confounders and to verify the reliability of the findings. Furthermore, the study did not analyze the metabolic pathways of the SDMs, making it challenging to speculate whether the SDMs contributed to inflammation and immune responses leading to disease. Future studies may benefit from metabolomic analyses to elucidate these potential mechanistic links. Lastly, the SHAP results were not experimentally validated, limiting the study’s conclusions. Further studies should aim to experimentally validate the SHAP results and improve the credibility of our findings and their applicability in clinical practice.

## Conclusion

By comparing the composition of intestinal flora in SLE and NC groups, this study showed that the alpha and beta diversity of intestinal flora in SLE patients were significantly different from that of NC individuals, which suggests dysregulation of intestinal flora in SLE patients. Additionally, this study revealed the characteristics of the intestinal microbiota in SLE patients. ML algorithms combined with SDMs can be used to identify SLE individuals, especially the XGBoost algorithm, which facilitates SLE prediction and provide a reference for clinical decision-making. Moreover, SHAP analysis helps to identify the relationship between SDMs and the risk of SLE, which provides valuable information for improving scientific treatment for SLE in the future.

## Data availability statement

The data presented in the study are deposited in the NCBI repository, accession number PRJNA1045348.

## Author contributions

WS: Writing – original draft, Conceptualization. FW: Formal analysis, Writing – review & editing. YY: Writing – review & editing. YHL: Supervision, Writing – review & editing. QW: Validation, Writing – review & editing. XH: Methodology, Writing – review & editing. YFL: Funding acquisition, Investigation, Supervision, Writing – review & editing.
